# Oyster Care: An Innovative Palliative Approach towards SPMI Patients

**DOI:** 10.3389/fpsyt.2020.00509

**Published:** 2020-06-08

**Authors:** Ilse Decorte, Françoise Verfaillie, Loïc Moureau, Sandrine Meynendonckx, Kim Van Ballaer, Isabelle De Geest, Axel Liégeois

**Affiliations:** ^1^OPZ, Geel, Belgium; ^2^Gezondheidszorg Bermhertigheid Jesu, Brugge, Belgium; ^3^University of Antwerp, Antwerpen, Belgium; ^4^PZ Onze Lieve Vrouw, Brugge, Belgium; ^5^KU Leuven, Leuven, Belgium

**Keywords:** Severe and Persistent Mental Illness, palliative approach, Oyster Care, holistic care, mental health, psychiatry

## Abstract

Oyster Care is the result of the search by caregivers in Flanders, Belgium, to develop quality care for patients with a Severe and Persistent Mental Illness (SPMI). This article offers a conceptual analysis of the Oyster Care model, based on experiences, analysis, and reflection of the authors, and on several examples. The starting point of the development of this new care model is the complex and difficult context of the care for SPMI patients. Their needs and suffering are very challenging on account of a wide variety of causes. At the same time they are in danger of being neglected by the care system. Paradoxically, the development and implementation of psychosocial rehabilitation in Belgian mental health care puts the care for these patients under pressure. In practice, they are often exposed to over- or under-treatment. Another aspect that has influenced the search for more qualitative care in cases of severe psychological suffering in general and palliative approaches in particular is the background of the legal regulation of euthanasia in Belgium. Oyster Care is an innovative form of the palliative approach and philosophy, tailored to the specific target group of SPMI patients. The caregivers create an “exoskeleton” or “shell” in which SPMI patients can “come to life”: they are mainly dependent on the “external structure” they receive in order to function, rather than on the “internal structure” of their abilities. It is a dynamic approach that responds to the needs, possibilities and pace of each patient: within this safety, people can fold back or take new steps. Oyster Care is also a holistic care approach, based on four pillars: physical care adequately responding to the somatic impairments of these patients; psychological care changing the scope of therapy by focusing on mental comfort and wellbeing; social care providing a structure of daily activities and contacts; existential care enhancing the experience of life as valuable and meaningful. The wellbeing of patients is paramount and requires a range of interventions, such as a highly personal approach, a flexible dealing with rules, a great dose of creativity in everyday life, extensive expertise in somatic care, and specific attention to existential needs and the search for meaning. The development of this care model in a number of care units in Flanders increases the wellbeing of the patients and creates a significant positive dynamic among caregivers. However, more research and resources are needed to further develop and integrate this model.

## Introduction

Caring for patients with a Severe and Persistent Mental Illness (SPMI) is a major challenge. Woods et al. (1) define people with SPMI as patients suffering from a prolonged or recurrent mental illness, who are impaired in activities of daily living and who require long-term treatment. Most authors mention bipolar disorders, major depression, schizophrenia, and anorexia nervosa as common diagnoses (1–3). Other diagnoses have been noted both in the practice of caring for SPMI patients and in specialist literature: these are mainly personality disorders and anxiety disorders, but also chronic addictions and autism spectrum disorders (4). Most of the SPMI patients suffer from a combination of different disorders. As a result, patients’ problems are deeply complex in nature. They often exhibit disruptive, destructive or self-destructive behavior and have therapy-resistant, severe psychological and socially disabling symptoms. They have complex care needs in many areas of life. For these patients, the treatment options are limited or do not even seem to exist. Their psychological suffering is severe, and a substantial subgroup needs permanent and intensive residential care.

Caregivers observe that these patients are admitted repeatedly and for a long time throughout their lives. They get stuck in treatment and psychosocial rehabilitation efforts at an early stage. Often, these patients do not reach remission or recovery. In some cases, the development of the care relationship fails and the caregivers become demoralized (5). There is initially a risk of overtreatment due to the persistent continuation of unsuccessful strategies. Later in the care process, the risk of neglect arises: SPMI patients end up in care units with a lower supply of care, where investments in therapeutic support are more limited, the staff is less therapeutically qualified, and the infrastructure is less adapted (6).

The development and implementation of the recovery model and psychosocial rehabilitation have been important steps in mental health care. An interesting initiative that promotes recovery and psychosocial rehabilitation for people with SPMI is the Active Recovery Triad (ART): long-term intensive care with a focus on increasing involvement in daily life, reducing pressure and coercion, and offering a hopeful perspective (7). In this specific population with refractory symptoms, however, the usual approaches of recovery and psychosocial rehabilitation alone are often not sufficient to improve wellbeing. These patients need permanent residential support. It is difficult for them to find a satisfying and meaningful existence because of their suffering, their living conditions, and their extensive loss experiences (8).

The situation of some of the SPMI patients can, in a certain sense, be compared to cancer patients in the 20th century. Oncological treatments were no longer effective and patients suffered from the side effects like fatigue, nausea, joint pain, etc. Otherwise, there was therapeutic nihilism: there was “nothing” more that could be done. Palliative care originated in that tension between therapeutic stubbornness and nihilism. Initially, palliative care was aimed at patients in a terminal state. The novelty of the approach was to abandon the original goal of prolonging life, and to focus on quality of life. Palliative care has meanwhile evolved into an evidence-based medicine that nevertheless often prolongs life by aiming at quality of life. Under the current approach of the WHO (9), palliative care should not be linked *a priori* to terminal situations. Palliative care also addresses early-stage and non-terminal conditions in any chronic disease for which there is no longer specific treatment. The treatment is often not discontinued but there is a transition in which the focus gradually shifts more to care instead of cure.

The same applies to SPMI patients who have become resistant to therapies. On the one hand, caregivers run the risk of therapeutic stubbornness by continuing to offer the same treatments without effect. On the other hand, as a reaction to this stubbornness, they tend towards therapeutic nihilism by foregoing any further treatment. In this area of tension, however, there is an opportunity to do something new.

This tension prompted caregivers to develop Oyster Care in a number of psychiatric hospitals in Flanders, Belgium. The objective of this article is to introduce this innovative palliative approach towards SPMI patients. The article has the following structure. First we briefly sketch the development of the concept. Then we make a thorough analysis of the concept. Finally we formulate some conclusions.

## Development of the Concept

### Origin and Background

In Belgium, since the euthanasia law of 2002, people in a medically hopeless situation with persistent and unbearable physical or mental suffering that cannot be alleviated, can have euthanasia performed by a physician under certain legal conditions (10). This possibility confronted caregivers and researchers with the question whether there is a palliative response to unbearable psychological suffering.

In September 2011, the “Committee for Palliative Care and Psychiatry” was established within the Flemish Federation for Palliative Care. This Committee brought together both caregivers and researchers from the psychiatric and palliative world. The authors of this article belong to this Committee. They investigated the domains where palliative and psychiatric care meet. They explored not only the question of palliative possibilities in the case of a euthanasia request. They also paid attention to psychiatric patients who end up in a palliative and terminal situation because of a somatic or psychiatric disorder, such as addiction or anorexia nervosa. Finally, they also addressed the situation of SPMI patients who do not ask for euthanasia but who are suffering unbearably, appear to be resistant to psychiatric treatments and for whom the question of quality of life arises.

In the same period, in Belgium, in the context of the development of community-based mental health care and the recovery movement, psychiatric hospitals were encouraged to reduce the number of beds for long-stay patients and to invest in the implementation of recovery thinking, more ambulatory and community-based treatment, activation and better housing support for patients with SMI.

In addition to the ambition to develop care for unbearable mental suffering, the pressure of community-based mental health care with hospital bed reduction also played a role in the search of appropriate care for this specific target group, the most serious SPMI patients. The concern was that this target group would end up again back and forth, as formerly in the long-stay wards, where patients and their caregivers received too little recognition for their needs. They were now at risk of ending up on the street, which in both the Netherlands and in Belgium goes against a strong tradition of comprehensive residential care (11).

For a small group of patients with SPMI, the psychiatric problems turned out to be so important in their dysfunction and serious disturbing behavior, destructive to themselves and others, that reorientation towards community-based mental health care was not possible, so that permanent residential support seemed the only option. At the same time, the caregivers came across the fact that the infrastructure, care, and treatment were inadequate for this target group.

With this background, the practice of Oyster Care was developed simultaneously and initially separately in two facilities, namely in the OPZ Geel, a public psychiatric hospital, and in the OLV Bruges, the Notre Dame hospital. On the workplace, caregivers developed this care with the aim of providing high quality care for SPMI patients.

This care was first discussed at a meeting of the “Committee for Palliative Care and Psychiatry” in December 2013. In collaboration with members of that Committee, this care was elaborated and scientifically underpinned. This care approach was also introduced in other Flemish psychiatric hospitals. Their input allowed the concept to develop further. The model of Oyster Care as we present it now, exists for three years, but it is still evolving. At present, the authors have knowledge of four care units that work according to this model. These units currently care for approximately 120 patients with roughly 90 FTE staff members, whom often have lower therapeutic qualifications due to lower education level and less access to therapeutic training. A large number of other hospitals or care units are still experimenting or using elements of it.

Oyster Care is a translation of the palliative philosophy in psychiatry and adds palliative elements to the already existing recovery thinking. It is a holistic care approach that originated from the specific needs and requirements of SPMI patients who permanently need residential support. Like classical palliative care, it complements treatment options that are still useful, but focuses more on daily quality of life.

The concept of Oyster Care is actually the English translation of the Dutch word “schelpzorg” (“shell care”) or “crustatieve zorg” (“crustative care”). “Crusta” is the Latin word for “shell.” The translation to Oyster Care further refines the metaphor. The authors of this article are clinical experts and researchers who together have developed this concept based on their clinical experience and literature study. They have purposefully chosen a different name than “palliative care,” not least because they want to avoid unjustified negative connotations with end-of-life care, such as the loss of hope, a pitfall mentioned by several authors (3, 5, 12). First and foremost, they chose the new name to emphasize the specificity and uniqueness of this approach.

### Description of the Concept

The most important characteristic of the concept of Oyster Care is that the caregivers create an “exoskeleton” or “shell” in which SPMI patients can “come to life”: they are mainly dependent on the “external structure” they receive in order to function, rather than on the “internal structure” of their abilities. The exoskeleton can be found in the four pillars of Oyster Care.

The original inhabitant of the oyster is the mollusk. The pearl originates as a reaction to foreign objects that have invaded the oyster. In turn, the oyster resists what it considers a threat by hardening the foreign object. The pearl is, therefore, the result of a coping mechanism of the oyster. In SPMI patients the original coping behavior fails and the fragility of their person is threatened. In Oyster Care, the shell closes around the fragile patient and its pearl. The caregivers accept the patients with their aberrant behavior, value their coping mechanisms as a pearl and protect them against harmful behavior. In times when the care needs are greater or when the patients’ behavior and decisions cause disproportional harm to themselves or others, the shell closes down further. At other times, when care needs are smaller and behavior and decisions are less or not harmful, the shell may open up more. The shell can be more open or closed, depending on the possibilities of enabling a meaningful life and on the necessity to reduce harm. The shell can usually not be removed. If this does happen, patients very often relapse into destructive behavior. The caregivers help patients to find their autonomy, but they take over if their decisions and behaviors are too harmful to themselves or others.

In addition to comfort care, the providers of Oyster Care offer a range of services in the field of care and treatment, recovery, and psychosocial rehabilitation, in which they support, adapt, adjust, and take over. Diagnoses are increasingly relegated to the background, and symptom treatment is foregrounded. In other words: Oyster Care focusses primarily on creative ways to deal with symptoms and the suffering they cause, rather than on diagnoses. Therefore, it is important to note that Oyster Care only begins after the completion of other stages of care, in which much critical attention has been paid to diagnosis and re-diagnosis. In Oyster Care, symptoms are not primarily understood from these diagnoses, but from the whole person. That means that the motivation to treat a symptom is the extent to which the patient suffers from it. Caregivers first try to understand the symptom and behavior: from where does the patient express this symptom, what meaning is hidden behind it, what function does it have in the whole of his personality? Oyster Care wants to cause as little damage and suffering as possible, both for the patients and for their environment. Support in case of loss and mourning, in all their aspects, is also part of this approach. The aim is to relieve the pressure of suffering, to help bear the burden of life, to treat the symptoms optimally, to limit the side effects of pharmacotherapy, to preserve and restore human dignity, and to find ways to pass life in a meaningful manner. In order to achieve this goal, integrated care is needed, hence the expansion into the four pillars: the physical, the psychological, the social, and the existential.

### Positioning in Literature

In order to further elaborate this concept, we position it in the existing literature. To this end, the authors carried out a brief study of the literature. They started from a literature search in PubMed, combining the search terms “palliative psychiatry” or “palliative approach,” and “severe mental illness” or “severe and persistent mental illness.” Then they applied the “snowball method” to these documents to find other relevant titles.

This search made clear that the amount of literature specific to the palliative approach of SPMI patients is limited. Several authors see similarities between care for SPMI patients and a palliative approach. Lopez et al. (13), Geppert (14) and Trachsel et al. (15) discuss medical futility and whether or not to discontinue treatment of refractory symptoms in anorexia nervosa patients. Trachsel et al. (6) question the conceptualization of “advanced stage of illness” in psychiatry, by analogy in somatic palliative care. Trachsel et al. (6), Baldinger-Melich et al. (16) and Donald et al. (17) highlight the need to develop a palliative paradigm for SPMI patients and argue for the need to develop good practices, research, and training. Trachsel et al. (3) observe that there is a positive attitude towards a palliative approach for SPMI patients among Swiss psychiatrists, but that there is no consensus on what this palliative care model might encompass for that target group.

### An Example

We can further illustrate this description of Oyster Care by presenting some examples or “construed cases” throughout the article. These are pseudonymized cases that refer partly to real patients and are partly modified and constructed so that the identity of the patients cannot be traced and that they offer a good example of the practice of Oyster Care. Here we present the “construed case” of Liz.

Liz is 66 years old and has had several and increasingly prolonged admissions to various hospitals and wards in the last 14 years. For this period she has been an outpatient since the age of 18. She has been in the Oyster Care unit for three years. She was diagnosed with both “bipolar disorder” and “personality disorder.” Before she was admitted, she stayed at several hospitals and care units. She had behavioral problems and she was showing more and more regression. In several instances her muscles were cramped and she kept saying: “I can’t.” When the caregivers wanted to help her, she became aggressive. Cognitive-behavioral therapy did not seem to bring any improvement. She frequently fell for unclear reasons and suffered from fractures. Because she did not report any symptoms of pain, the fractures went unnoticed and were treated too late. Once the plaster cast had been removed, Liz refused to use her hands, resulting in permanent contractures. She resisted any form of help and refused to eat food or take medicine.

After another fall, Liz suffered a spinal fracture. The caregivers concluded that she should be protected by them. With Liz’s consent, they decided to restrain her for six weeks. This fixation took place during the day in the living unit. She was fixed in an easy chair. During this period, the team received an additional caregiver from the management, so that they could provide individual, one-on-one care. She was not allowed to walk, but the caregivers tried to connect with her and to fulfill all her other wishes as best they could. They succeeded in making her eat again. She was offered food whenever she wanted. She did not have to adhere to the usual care unit structure.

The first few days were difficult: Liz was over-excited and resisted the interference. Gradually, however, she began to accept the care and to communicate with the caregivers. Together, they tried to express her suffering in words: she did not know how to endure her crises and she had no insight into the origin and course of her behavior. By looking together for what was helpful to her, she felt treated as a human being again. For the first time in a long period, the caregivers could connect with a healthy, functioning part of Liz.

After these initial six weeks, Liz’s behavior had changed: she no longer fell, her worst behavioral disturbances had disappeared, as well as part of her suffering. Before the fall that led to her spinal fracture, she linked her self-esteem to her ability to make and execute decisions: “I am only a person if I can carry out my own decision”. She failed at this, which led to self-destructive behavior. After her last fall and treatment, her self-confidence changed: “I am someone because they take such good care of me. I am worth taking care of”.

These days, Liz still needs the unit’s Oyster Care to live well. For example, she needs extra supervision during meals due to swallowing problems. Individual contact often goes well. The repetitive behavior, however, sometimes manifests itself in repeated questions such as “what should I do now”? Together, Liz and the caregivers have drawn up a crisis intervention plan in which she gives consent to administer Olanzapine intramuscularly if she refuses her oral medication in times of crisis. Liz is still struggling to make decisions based on her “gut feeling.” This difficulty is why the caregivers help her: they offer her activities that they know she likes to do. For example, music therapy consists of singing together and gives her a feeling of wellbeing.

## Analysis of the Concept

### Physical Pillar

After the presentation of Liz’s example, we now proceed to a more extensive analysis of the specificity of Oyster Care, based on the four dimensions of palliative care. This care starts with the physical pillar. Patients with SPMI have many physical needs. Their life expectancy is about 20% lower than that of the average population (18). Important causes are an unhealthy lifestyle, side effects of medication, a higher incidence of diabetes, an increase in metabolic syndrome, with more cardiovascular risks, cancer, chronic obstructive pulmonary disease, etc. Stigma and the high threshold of health care play a role in the lower life expectancy. An additional specific issue is that people with SPMI are more likely to die from choking or asphyxiation (19). Abnormal eating activity, as a result of behavioral changes inherent in institutionalization and in the psychiatric problems themselves, increases the risk of suffocation (20). Behavioral changes such as hyperphagia and tachyphagia (fast-eating syndrome) are considered to be the most common causes of asphyxiation and aspiration (21).

A major challenge for caregivers is the different bodily experience for these patients, often resulting in a different pain experience (22). For instance, important diagnoses such as fractures or cancer are recognized too late because patients can tolerate a lot of pain and cannot communicate quickly or do not emotionally show their suffering to others. Even for physicians who have years of experience with patients with SPMI, it remains difficult to make a correct and timely diagnosis (16). An additional problem is that often, when making a diagnosis, not all examinations and treatments are possible because of psychiatric problems, for instance insufficient cooperation due to behavioral problems or difficulties in following instructions. There are also ethical dilemmas, such as the difficult assessment of the patients’ decision-making capacity when treatment is refused due to a lack of disease insight by the patient (2).

In line with palliative care, it is important not to approach the treatment of pain in a strictly biomedical way. This means that pain is not considered a purely somatic fact, and that the influence of psychological, social, and existential factors on the experience of pain is also taken into account. Usually, this “total pain” is not relieved by only classic painkillers; other approaches are needed as well. Oyster Care argues for the further integration of the somatic and psychiatric aspects into care.

Liz’s example can help to further clarify the complex relationship between somatic and psychological functioning. Liz frequently fell for unclear reasons and suffered from fractures. Because she did not report any symptoms of pain, the fractures went unnoticed and were treated too late by the caregivers. Once the plaster cast had been removed, she refused to use her hands, resulting in permanent contractures. The physical damage she suffers by not expressing her pain is considerable. Moreover, the cause of the falls is likely to be the result of a psychiatric disorder rather than a somatic cause. This is evidenced by the fact that the physical examination for the causes of the fall was unsuccessful and that the falls stopped when the psychiatric illness was cleared.

As far as the activities of daily life are concerned, much more is usually taken over by the caregivers than is strictly required by the physical condition. Only short-term appointments are made. They reflect the patients’ mental condition: if patients feel that they are able to carry out the activities, they are encouraged to do so, but this engagement can be very variable depending on the circumstances. At times when the psychiatric disease process escalates, the symptoms may be so prevalent that the patient can no longer adhere to the appointments. Then new appointments are made that are adapted to the current capabilities of the patient. In the eating environment, special individual attention is paid to the risk of swallowing. For all these reasons, one-to-one accompaniment in these care units is recommended or even essential.

In the example of Liz we see that during the escalation of her illness, she does not have to stick to the structure of the care unit, and she can eat whenever she wants. Once she is better, new appointments are made. Liz, too, has had several swallowing accidents. Eating is done under supervision. Liz needs the external structure of the care unit for her physical safety. The caregivers have to make sure that the falls can be prevented as much as possible and that the fractures are noticed and treated. They also make sure that she does not choke. There is much more need for intervention by the caregivers than in other units. The care unit works like a shell: it ensures as much safety as possible in order to preserve physical integrity as well as possible.

The treatment of patients with SPMI is focused on wellbeing and optimal symptom treatment. These patients need continuous psychiatric clinical follow-up. Traditional therapy guidelines, developed on the basis of acute psychiatric disorders and long-term care after stabilization, are often not sufficient. Psycho-pharmacotherapy is reaching the limit of therapy resistance. Unfortunately, there is no research into pharmacotherapy in therapy resistance. Due to the use of co-medication or due to a lack of cooperation, SPMI patients are usually unable to participate in research regarding the use and effectiveness of medication. Consequently, guidelines remain silent on the topic of therapy resistance. Pharmacotherapy, on the other hand, is in the Oyster Care model a continuous, tailor-made balance between careful symptom treatment and limiting side effects, designed in order to alleviate suffering, increase general physical and mental wellbeing, and influence behavior, so that it becomes more livable for both patients and their environment. Sometimes prescribing off-label medication can be useful for symptom-control. In any case, it is necessary to ensure a structured evaluation of pharmacotherapy based on existing or individually developed measuring instruments.

In order to achieve this physical care, there is need for many more caregivers with somatic expertise than in other care units. It is necessary that the general practitioner is part of the care team as well as nurses with sufficient somatic knowledge and experience.

### Psychological Pillar

In the same way as the palliative care approach, Oyster Care aims to respond to the vulnerability and poor protective capacity of people suffering from severe psychological problems. The focus is not primarily on remission or recovery, but on making this suffering more bearable.

The cooperative relationship between patient and caregivers is in Oyster Care also a cornerstone. Patiently and persistently building up a relationship of trust can be a challenge, given the seriousness of the psychiatric disorder and the basic mistrust these patients experience in their social contacts. Many patients have failed to build trusting relationships because they have suffered a lot of relational damage, have little or no trust in other people, or have not developed any secure attachments.

Given this delicate context, caregivers need excellent therapeutic attitudes and communication skills all the more, to build up a relationship of trust and to develop a social connection. From the patients’ perspective, the caregivers ensure safety and structure. By applying the psychoanalytical concepts of “holding” and “containment” and taking an “emotionally available” position (23), the caregivers create a sufficiently safe “shell” in which connection becomes possible. With the help of this “shell,” patients can find and maintain an appropriate level of stimulation. They are protected against external and internal over-stimulation, such as anxiety or severe mental distress.

In the example of Liz, the elements of “holding” and “containment” are especially important after her vertebral fracture. The first few days were difficult. She was over-excited and resisted the interference. Gradually, however, she began to accept the care and to communicate with the caregivers. During the first week, it took the caregivers a lot of effort to restore the therapeutic relationship and regain Liz’s trust. Once the trust was revived, Liz began to reconnect with the caregivers. Paradoxically, the recovery of the relationship did not happen despite but thanks to the fixation. As a result, she was no longer able to push the caregivers away and avoid providing care. The purpose of the fixation was to prevent an unstable vertebral fracture resulting in a paresis of the lower limbs. But in those six weeks of fixation a therapeutic relationship could be established again. The fixation worked as a kind of border that provided protection (containment) and that allowed the caregiver to be available in a reassuring way (holding).

The ideas of Tielens and Prouty are important sources of inspiration for building supportive contact with patients who lack contactual skills. Tielens (24) advanced motivational techniques to connect with patients with severe psychosis, especially when they lack self-insight. Prouty et al. (25) developed a pre-therapy model that is part of the phenomenological tradition in psychology and psychiatry with its origins in the experiential psychotherapy of Rogers and Gendlin. The aim is to restore three natural contact functions, namely reality contact (perception of the world), affective contact (awareness of moods, feelings, and emotions) and communicative contact (giving meaning to observations through language). This model can be used for various indications such as catatonia, auditory hallucinations, dissociative identity disorder, dementia, etc. The therapist uses contact reflections that are empathically attuned to the elementary level of the patient: naming the situation, event, facial expression, body posture, repeating word for word, making reiterative reflections to perpetuate the contact, etc. In this way, the patient is helped to re-establish contact with everyday reality and to come out of his psychological isolation. Contact reflections in group about concrete events such as the news, the weather and extra activities can also be used to strengthen the fragile contact with reality and the others.

Oyster Care is also inspired by the concept of “woodshedding,” a term used by American jazz musicians and in line with the concept of moratorium, a waiting or rest period needed to develop something new. In this “woodshedding” phase, the patient runs the risk of getting stuck in understimulation, in isolation or in overly high expectations, and failing to respond to contemporary models of brief psychotherapy. Caregivers are investigating whether further therapeutic process that can contribute to a more qualitative life may be initiated with these patients (26, 27). They continue to invest in the development of a supportive clinical psychotherapeutic treatment environment. The therapy is not aimed at “change” or at “gaining insight” beforehand, but at creating the right conditions for contact and for relieving suffering. It wants to increase the wellbeing in the present by creating a sense of welfare. In the example of Liz, music therapy gives her a feeling of “well-being.” She looks forward to it and enjoys it, but it gives her no further insights and does not contribute to a “healing process” by curing the mental illness, but by reducing the burden and suffering.

In Oyster Care, different therapeutic frameworks are used, and both verbal and non-verbal treatment methods are applied. These are attuned to the patients’ perspective and pace, to the seriousness and nature of their psychological vulnerability, to their cognitive capacities and their possibilities for introspection and mentalization. It is an important task for caregivers to creatively explore how existing frameworks and psychotherapeutic methods can be fine-tuned for this patient group. The therapy should not be continued or discontinued, but instead adapted to the target group. The therapy aims to increase wellbeing in the present by creating a sense of welfare. The therapy is not primarily insightful, but creative. Reducing the burden of suffering is the most prominent aspect.

Together, the chaplain and the psychologist of an Oyster Care unit developed “the Story out of the Closet.” This approach is inspired by the Japanese narrative theatre, or “kamishibai,” as well as by pre-therapeutic and narrative techniques. By reading a story, that fits in the interest and life world of the patients and by using picture books and coloring pages, patients are invited to talk, either about the story they read, or about their own story. These sessions not only help the caregivers to get closer to the patients’ life world, but also the patients themselves keep in touch with their own life story and identity. They can help to discover wishes, interests, needs, which can lead to the improvement of the quality of life and to appropriate activities. Reading and telling activate concentration, memories, and imagination, makes room for questions of meaning and can bring people together, help to make contact and break through social isolation.

Patients are often unable to think and speak about themselves in linguistic concepts. Usually, they do not have a coherent and structured sense of “I.” The experience of oneself in the here and now can also be absent. In the treatment, a good understanding of communication styles and of the meaning of verbal and non-verbal signals is important (28).

The relationship of trust comes under pressure at a time when patients, due to limited self-insight and reduced decision-making capacity, are making choices that may harm themselves or others. As a result, “shared decision-making” is often no longer feasible. Caregivers frequently find themselves in an ethical tension between guaranteeing safety and offering wellbeing. “Positive risk-taking” is a crucial element: shared responsibility when this is possible, and when it is not possible, taking over responsibility (29). Positive risk-taking means that we always try to give patients the benefit of the doubt. This approach has been developed in Bowers’ Safewards Model, which contains many inspiring elements for dealing with aggression and restriction of freedom (30).

It is important to reduce as much as possible coercion and other forms of interference. If coercion is applied, it is done under strict conditions that can be motivated and evaluated, both with the patient and in the team. Ethical consultation can help to thoroughly reflect on the justification of restrictions of freedom and of coercive measures (31). Regular supervision and intervision moments among the caregivers are essential as well.

In the example of Liz, caregivers take the risk of falling as long as there are only fractures of the hands. They consider her to be autonomous and are limiting freedom-restricting measures. In the case of a vertebral fracture, the risk becomes too great and they decide to intervene. On the evening of her fall, she is mentally sound and aware of the risks of her own behavior. She admits that she cannot control herself at her “bad moments.” She realizes that she is hurting herself but says it is stronger than herself. At that moment, she gives her consent for the fixation. But in the following days, she resists and withdraws her consent. At that moment, she is less autonomous: her thinking is not coherent, she denies the reality in which she finds herself, and her decisions do not correspond to the values in her life. She is a proud woman who has always taken good care of her body during healthier periods. Not only do caregivers fix her in order to protect her physical integrity and prevent further damage, they also base their decisions on the conversation they had with her at the moment just after her fall.

This is an example of how caregivers deal with autonomy in SPMI patients: they do not always follow the patients in their daily decisions, especially when the damage they inflict on themselves is too great. On the other hand, caregivers always look for the underlying autonomy of the patients: who are they, what are the values they want to respect and protect, and how can they protect them at times of less autonomous decision-making? This is what caregivers see as an important characteristic of Oyster Care. Liz and the caregivers have together drawn up a crisis intervention plan in which she gives permission to administer Olanzapine intramuscularly when in times of crisis she refuses oral medication. In order to guarantee the autonomy of the patient as much as possible, caregivers also use signaling plans. In the next example, we show how caregivers can deal with autonomy and risky behavior.

Bert is an autistic man of 52 years old with severe behavioral disorders. He picks up cigarette butts from the ground and eats them. He also drinks large amounts of coffee all at one time and spills a lot of coffee on the floor. As a result, the kitchen is covered in coffee. This happens not only in the kitchen of the care unit, but also in other coffee rooms in the building. No therapy or medication has any influence on this behavior. Freedom-restricting measures lead to a serious loss of wellbeing and have hardly any effect: this makes his behavior more destructive and aggressive. Ultimately, freedom-restricting measures were stopped and Oyster Care was started. The caregivers choose to adapt the environment to Bert instead of Bert to his environment. They give him a limited supply of “clean” cigarettes to eat and he gets his thermos of coffee. They arrange for him to drink his coffee with milk, otherwise, he burns his throat. He gets his part of the care unit’s kitchen where he can spill coffee. The other coffee rooms in the rest of the building are locked. The caregivers have had to plead for this agreement with other staff members. These measures already make life more bearable for Bert, but the caregivers are searching further. Because it seems that Bert is looking for coffee to calm down, they suggest methylphenidate. This medication is effective: the urge for coffee and cigarettes has been greatly reduced. Nevertheless, from time to time he still eats his “clean” cigarettes.

Bert is an example of how Oyster Care works in practice. The aim of the interventions is no longer to remove his disruptive behavior so that he can live in society again, but to create an environment in which he can exist with his deviant behavior. The caregivers strive for the highest possible quality of life for Bert and try to limit the suffering for him and his environment as much as possible. Safety is not lost sight of, but is less of a priority. It is an example of positive risk-taking. When dealing with Bert, caregivers mainly appeal to their creativity and out-of-the-box thinking. Non-working medication and therapies are stopped, but the search for what does work continues. An example of this is experimenting with off-label medication, as is the case with methylphenidate.

### Social Pillar

The example of Bert is a good introduction into the social pillar: the example makes clear how caregivers bridge the gap between the care of an individual patient and the organization of a care unit in a hospital. In an organization, rules and regulations are always needed to make good living and good care possible. However, these rules are always of a general nature that cannot take into account the individual situations of patients. This is why caregivers need professional freedom to deal with these rules in a flexible manner (31). They must be able to deviate from the rules when it is conducive to the wellbeing of an individual patient, without harming the interests of other patients.

Due to their long history of hospitalization and illness, patients’ relationships with relatives have usually been damaged or lost. Patients often have a limited social network and are confronted with loneliness. Contact is frequently limited to fellow patients and caregivers. The hospital and the care unit have become their home: the relationship between caregivers and patients is very important and meaningful. Despite the serious difficulties experienced by patients in terms of communication and social contact, caregivers help them to reconnect with others. After all, relationships are an important source of meaning in life (32). Moreover, by connecting with others, patients can connect again with themselves (33).

In daily life in the care unit, the caregivers create a reliable environment or “exoskeleton” which offers opportunities for encounters and where being “strange” or out of the ordinary is recognized, respected and tolerated. The caregivers are as tolerant of “strange” behavior as possible as long as the situation remains safe for all people concerned.

Patients need caregivers in order to create a reliable environment and meaningful and safe contact with others. Caregivers provide recognition and predictability for daily recurring activities by establishing fixed anchoring moments throughout the day program. They remain close by in a tactful way and are available to provide assistance when contacts risk running aground (28). In addition, their social behavior is a model for patients (34). In Oyster Care, these different roles and functions of caregivers are of vital importance and are fulfilled flexibly, according with the vulnerability and possibilities of patients. There is a special bond between caregivers and patients that leads to a different dynamic of distance and proximity. The caregivers are sometimes the closest to patients, especially when there are no relatives (8). The dependent position of the patients creates a demand for more proximity. This demand comes from the patients who have the desire to enter into a relationship, but also from the caregivers who willingly assume this role due to their empathy. This can be a pitfall for caregivers: because of their position as significant others, they sometimes assume the role of family. This increases emotional involvement and allows them to get into trouble when making certain decisions that require a more professional distance. Here as well, supervision and intervision are essential so that caregivers can attain an appropriate balance of proximity and distance.

Relatives, together with patients, have often gone through a lengthy treatment process. They continue to struggle with the condition of their loved ones and still hope for improvement (35). Caregivers continue to invest in caring for the family, by acknowledging their often lifelong commitment and by providing them with emotional support and information. Family and relatives are also involved as much as possible in the development of care.

For many SPMI patients, contact with society has been interrupted. They do not want to participate actively and stay “inside” the hospital because it hurts “outside” (33). They feel alienated and often rejected by the outside world. The care unit has become their living world and feels familiar. The structure of the unit is as a shell that offers them security since they are familiar with the expectations and the rules. The caregivers do not just strive for the normalization and socialization of patients. Rather, the caregivers support the patients’ desire to belong and to live in a meaningful way, fighting against processes of marginalization and exclusion, both within the care unit and the hospital and in the outside world. As a “skilled companion,” caregivers form the bridge between patients and the world around them (36). They organize life at the care unit and in the hospital and strive for a place where patients feel at home. They support patients in taking on and maintaining roles other than that of “patient,” by being present in their lives and undertaking activities together (37). In Oyster Care, this is done in the safe context of the hospital, the immediate neighborhood or in the trusted circle of friends and family. The importance of a flexible approach to rules in the care unit and the hospital is illustrated by the next example.

Remy is a 69-year-old man diagnosed with an obsessive-compulsive disorder. He was admitted for the first time to the psychiatric hospital at the age of 20 years. Afterwards he can maintain himself in a very protective family environment. At the age of 40, after a serious brain injury, he develops also a hoarding behavior that becomes increasingly extreme with a lot of social nuisance, which increases even more after the death of his partner. This leads to a forced admission to the psychiatric hospital. The caregivers fail to develop a therapeutic alliance with him, neither psychotherapy nor psycho-pharmacotherapy brings relief and he is referred to the Oyster Care unit. Here Remy continues to exhibit a very disturbing behavior in the care unit: for instance, smuggling food leftovers into his room, due to a strong and exaggerated concern for the environment. He was also often verbally aggressive towards some caregivers. Despite their efforts, he managed to hide food remains in his room and other places. This led to a decrease in personal hygiene, an increase of odor nuisance and even a vermin infestation. In consultation with Remy, the caregivers turned to a “closed-room” system. They also set up an ethical intervision with the focus on freedom-restricting measures for Remy. They decided that a creative therapist, with whom Remy has good contact, would work intensively one-on-one with him. They also called on volunteers to take Remy on long nature walks. Since then, his aggression and disruptive behavior have decreased dramatically.

The example of Remy highlights the need for solidarity, allowing more people and resources to be allocated in exceptional situations so that one-to-one care can be provided. These resources may come from other care units within the hospital, or from (temporary) recruitment of additional staff. It is also possible to choose to work with fewer patients on the ward for a certain period of time. Of course, this approach requires good coordination with the management and the other care units. The importance of ethical deliberation and intervision in difficult situations is also evident.

### Existential Pillar

SPMI patients experience many losses in different areas of life. They suffer both from the disease itself and from its consequences. It is exactly in the sharp confrontation with the fragility and brokenness of existence that the usual, intuitive experience of meaning in life is endangered. The powerlessness in and meaninglessness of life then express themselves in the patients’ words and behavior. Patients often no longer cherish particular hopeful goals in their lives (“espoir”), but that does not mean that a fundamental attitude of hope in life is no longer possible (“espérance”) (38).

In line with the palliative approach, it is of great importance that caregivers within Oyster Care have the necessary competencies to recognize the existential needs and spiritual sources of patients, and to work in a supportive manner (39). “Existential” refers to the basic need to seek and discover meaning and purpose in existence, while “spiritual” refers to the experience that meaning in life can be found in the connection to a spiritual or transcendent reality. The generally accepted definition of spirituality in palliative care states that “spirituality is the aspect of humanity that refers to the way individuals seek and express meaning and purpose and the way they experience their connectedness to the moment, to self, to others, to nature, and to the significant or sacred” (40).

It is an essential task of all caregivers to pay attention to these existential and spiritual needs. When faced with the severe loss experiences of patients, they should address these questions and remain present in spite of their own powerlessness. Care units therefore need an atmosphere of “caring for caregivers”: they themselves have to learn to deal with permanent powerlessness as well. The “presence theory” of Baart (41) rooted in care ethics, with focus on interdependence and connection, human vulnerability, and responsibility, helps caregivers to approach patients in their suffering and to stay close to their world and perspective with an open, not only actively searching, but also a waiting, accepting, wondering attentiveness. This listening and attentive presence to the existential needs of patients is essential for all caregivers. However, when they meet spiritual questions that are rooted in a particular religious tradition, they can call on representatives of these religions or churches to guide patients in their religious practice.

We experience in the SPMI patients a strong will to live. Despite the legal possibilities in Belgium, people with SPMI seem to have a negative attitude towards medical assistance in dying (2). However great the suffering may be, the demand for termination of life is usually absent or remains in the background. These patients still want “to be there.” Recognizing the power of the will to live is very important. Patients often experience hopeless, almost unbearable and irreversible suffering, but they tend to endure it.

In the existential pillar, the shell forms an external structure to help the patients discover meaningful day activities. Caregivers help patients to experience meaning or purpose when offering activities. The shell also plays an important role in connecting patients, both with themselves and with others. Due to the long-term nature of their illness, SPMI patients are often no longer interested in and motivated to find activities. In the search for a meaningful existence, it is important to support them in finding, practicing, and maintaining activities. They still need to connect with themselves, with others, with nature, and with the meaningful and sacred in their lives. Often, this connection comes from the problematic part of their personality. By “problematic part” we mean the part of them that is responsible for their suffering and that is influenced by psychiatric illness. It is therefore up to the caregivers to continue to invest in connection: in the care relationship they try to connect with the remaining healthy functioning part of the patients. At the same time, this relationship serves to acknowledge the existence of patients (8).

The core of Oyster Care is searching in words and deeds for the essence of the human person, for the remaining seed of meaning and self-esteem, and encouraging them to grow. Caregivers actively seek out what patients still recognize of value in themselves. Very often SPMI patients cannot name this. They need the external structure of the care unit as a shell in order to identify and name values and meaning and to experience these values and meaning again in their daily functioning. The caregivers help the patients to make choices in which these values are expressed. They try to take a critical look at their actions and ask themselves what value they are trying to protect in every intervention. Do they stand up for their own values and standards or for those of the patient? The answer is not always clear. They are guided by “well-being.” This intervention should contribute to feeling “well,” of increased quality of life on the one hand, and to the feeling of being fully accepted as a “being”, a person on the other. The following example illustrates the importance of connections in existential care.

Christine is a 55-year-old woman with a complex psychiatric history. Since the age of 18, she has had many long-term and recurrent admissions to various psychiatric hospitals. Several diagnoses were made, including mood disorders, compulsive disorders and anorexia nervosa. Her history also bears witness to fugues and psychotic thoughts. There also seems to be a general deterioration. At that moment she is transferred to the Oyster Care unit. The caregivers have little real contact with her. Limited communication is only possible during walks. During those walks she says that she is lonely and unhappy and does not want to continue to live. She constantly takes objects from the living room and steals them from other patients as well. She brings them to her room and often destroys them. It seems as if the objects she takes out of the other patients’ rooms are not randomly chosen, but are important for that particular person. By doing this, it looks like she is connecting with others in a very negative way. She had a traumatic childhood during which she was abused. As a young girl, she also had to hand over the money she earned to her father. For Christine, relationships with others were certainly not positive: a relationship to her meant taking something away from the other.

Temporarily the caregivers reorganize themselves so that they can offer one-on-one care. Because someone is standing next to her all the time, they prevent Christine from stealing objects and help her to come to activities that give her a sense of meaning. She loves to dance, clean and draw. Through these activities, the caregivers teach her to connect with people in a meaningful way and to discover meaningfulness in daily life. This approach helps as long as they offer one-on-one care. However, because of structural limitations, they cannot keep up this system. At the moment, Christine no longer complains about loneliness. She also says less frequently that she is unhappy. She doesn’t want life to end. Her stealing has been reduced, but it has not disappeared altogether. She continues to need the caregivers to start her activities and can only be alone for about 10 minutes at a time. Then she needs her caregivers again to help her start another activity. This way she can have a short conversation with them and sometimes she can enjoy both her relationship and her activity again.

In this example, Christine needs the shell to connect. However, she connects with others from her clinical picture: she takes something meaningful away from the other and appropriates it for herself. This seems rather compulsive, and she has no control over it. The caregivers prevent her from stealing. They search with her for activities that are or once were meaningful, and they help her to start and carry out those activities. In this way, Christine learns that relationships with others also have positive sides. Thanks to the caregivers, she comes to activities that give her meaning. So the caregivers teach her to connect with other people but also with herself in a different way. She learns to enjoy activities and her relationships with others again. This reduces the pressure of suffering and increases her quality of life. In this example Christine permanently needs the caregivers to start her activities. So she constantly requires the external structure of the care unit to be able to connect with herself.

Also in the example of Liz, we see that caregivers are teaching her the positive aspects of a relationship anew. By continuously offering care during the period that she was fixated, she learned the benefits of the relationship with the caregivers. After the six weeks of fixation, she was able to re-establish a relationship that was normal for others and meaningful to her. There is no more aggression. The connection with herself has also been restored: she regularly dresses neatly, she no longer falls and no longer displays destructive behavior towards herself. At the moments of escalation of her illness she still shows regressive behavior. Then she needs the external structure of the care unit and the guiding presence of the caregivers more. At that moment, the shell is more closed. Liz continues to need the external structure of the care unit in order to achieve meaningful activity.

Some time later, Liz gets breast cancer. She chooses amputation, while oncologists recommend breast-saving surgery and ten rounds of radiotherapy. Her decision to opt for the amputation was based on her illness: “I can’t do it.” In conversations, the caregivers try to persuade Liz to opt for radiotherapy after all. Because of her psychiatric illness, she has already disfigured her body. Her hands are still in contracture from the earlier fractures. The caregivers remind her of her pride. They reassure her that they will take care of everything and that they will take over when she experiences that “she can’t do it.” They convince her through daily conversations in which they remind her of who she was and what she finds important at times when she feels better. In the end, Liz is persuaded and chooses the breast-saving surgery and radiotherapy. Afterwards she is very satisfied with this and thinks she has made the best choice: a choice Liz does not perceive as imposed to her, but very much her own.

It is necessary to think outside the box. Once it is clear that fixed protocols and guidelines do not work, a large dose of creativity is required to continue to invest in connecting with the patients. Sufficient effort and research will certainly be needed in the future to gain a better understanding of how caregivers can make that connection and pay attention to values and meaning in life. Although attention for the existential dimension has increased, it is certainly not enough structurally anchored in health care, and it remains underexposed. In the case of SPMI patients, who have just been confronted with hopelessness, powerlessness, loneliness, and meaninglessness, this dimension is of the utmost importance. It cannot be lacking in integrated care that puts their wellbeing first.

## Conclusions

Within mental health care, there is a group of patients who are in danger of being forgotten by the care system, the research community and society in general. Oyster Care aims to focus attention on this group, to identify their needs and to answer the question of how caregivers can improve their wellbeing and how to reduce the pressure of suffering: a form of holistic care, created by analogy with the palliative approach.

To make this possible, the caregivers for SPMI patients in Flanders advocate the further development of Oyster Care units, in analogy with palliative care units, with an appropriate infrastructure so that all aspects of life, wellbeing, care, and treatment come together. These are intensive, residential treatment units for long-term care and treatment with single rooms and preferably small housing units. These differ from the existing units in that patients are approached in a more thorough way from an integrated care perspective: attention is paid to the four proposed pillars of the palliative approach and their mutual relationship. Now we will make a synthesis of what is innovative in Oyster Care, sketch the initial experiences, point out limitations and formulate recommendations.

### Innovative Nature

In the first place, Oyster Care pays more attention to the integration of the somatic pillar of care. To guarantee this further integration, sufficient knowledge of the somatic problems in SPMI patients is required. It is therefore important that the general practitioner, together with the psychiatrist, forms part of the multidisciplinary team. Symptoms are interpreted in their totality: each symptom can have a physical as well as a psychological origin. Caregivers are more aware that SPMI patients may have a different way of expressing symptoms. In addition, there is more attention for activities of daily life, especially around eating. Psycho-pharmacotherapy puts the focus on continuous, tailor-made balance between careful symptom treatment and limiting side effects, there is also more space for off-label medication.

For the psychological pillar, the care relationship is even more important and often more challenging than in other care approaches. Therapy is not continued or discontinued, but is again translated to the target group. The therapy aims to increase the wellbeing in the present by creating a sense of welfare. The therapy is not primarily insightful, but creative. The role of positive risk-taking is crucial. Caregivers often deal differently with the autonomy of patients: they look for the remaining autonomy and try to protect it. They accept the patient with his abnormal, often disruptive behavior but prevent him from causing serious harm to himself or others. They consider him to be less autonomous and intervene. Reducing the burden of suffering is paramount.

In the social pillar, the caregivers have a more multidimensional role than in most other care units. They act as a bridge to the world and are important role models to the patients. The caregivers create a shell within which and through which encounters can take place and be stimulated. In order to guarantee freedom and safety as much as possible, an adapted housing structure with sufficient personal space and sufficient staff is required.

Finally, the existential or spiritual pillar is gaining in importance in this approach: to improve wellbeing, the search for a connection with the patients’ healthy part of their human “being” and thus the growth in “meaning” is central. The patient needs the external skeleton of the care unit in order to perform meaningful activities. Therefore, a sufficient number of caregivers in these care units should have the competences to deal with these underlying existential and spiritual processes.

### Initial Experiences

The initial experiences with the Oyster Care model in Flanders are encouraging. Aggression incidents and self-mutilation have decreased, and transfers to other care units because of unmanageable behavioral problems have become highly exceptional. Medications can be phased out, albeit to a limited extent, with a positive effect on the burden of side effects. There are fewer and fewer fatal swallowing incidents. A number of patients may indicate that they feel better despite their illness and suffering. Others, who are unable to communicate this development, feel an improvement in wellbeing, but their wrestling with the suffering remains tangible. Perhaps the most important achievement is the awareness of not being abandoned and the experience of belonging. Relatives indicate that they are more involved and that they feel recognized. They see a positive development in the patients.

Oyster Care also has positive effects for caregivers. In the past, these care units were often seen as “end stations” in which little was invested, for instance in psychotherapy, recovery, and training of caregivers. Oyster Care’s approach is now creating a positive appreciation and identity for the target group of SPMI patients and for the caregivers. Job satisfaction has increased, and the risk of burnout and compassion fatigue has clearly decreased. Working in the Oyster Care unit is seen as a challenge and opportunity, rather than a burden. There is more positive feeling of identity and less feeling of powerlessness. They experience satisfaction because they can let go of the therapeutic stubbornness without giving up the patient. Being allowed to think out-of-the box makes the work more challenging and personal. A similar dynamic was observed in caregivers working for and developing the emerging Partners in Recovery program (PIR) for people with SPMI in Australia (42).

### Pitfalls and Challenges

Here we sketch a number of pitfalls of Oyster Care that at the same time are challenges to prevent them. The most important pitfall and challenge lies in the moment when the caregivers start the Oyster Care. As already described, it is very important that they first pay sufficient attention to diagnosis and re-diagnosis. They must have tried and maintained therapies for a sufficiently long time before they can decide that therapy resistance is the case. Also during the provision of Oyster Care, caregivers need to be sufficiently aware of new therapeutic and scientific developments.

There is also an ethical pitfall, particularly the tendency to take over responsibility, which can be seen as a form of paternalism. However, this care does not have to be paternalistic if the caregivers discuss the care in advance and request consent as much as possible, if they remain attentive to all verbal and non-verbal signs of permission or refusal on the part of the patients, and if they involve the relatives in order to obtain a substitute consent (31).

In the existential pillar, caregivers look for what the patient recognizes as meaningful and valuable in themselves. They try to stimulate and shape the meaning and values in daily functioning and in the choices the patient makes. It is certainly a pitfall that caregivers prefer to advocate their own meaning and values instead of those of the patient. Sufficient critical and permanent reflection is needed. More research and enough people who are familiar with existential processes are paramount.

Additionally, the residential character of the model can be questioned as a pitfall. Current Oyster Care practices are about SPMI patients who have such a dominant psychiatric disorder that a return to society seems impossible. The behavior this target group exhibits is too disruptive to themselves or others, and the harm they would inflict on themselves or others would be too significant. The Oyster Care model and its residential approach are, of course, specific to the extensive residential network of psychiatric hospitals in Belgium. In other countries, there may be a different approach to this problem.

The caregivers are aware of this situation and sometimes want to open the shell a little more. This happens when the quality of life is increased, the connection with oneself is better restored, and thus choices are made that are more in line with the patient’s own values. So the caregivers open the shell when the autonomy is increased and there is less destructive behavior. In that case, other forms of living and care are creatively sought, in collaboration with community-based mental health care teams or sheltered home teams. The caregivers of these teams then share the Oyster Care approach in the care setting also outside the care unit, together with the team that offers day treatment. This often works to a limited extent, but the patient will usually revert to his former behavior once the shell has been opened to a large extent. Discharge procedures from the care unit often fail and those patients are then readmitted. In this regard, a pitfall for the caregivers is certainly to be sufficiently open for those moments when the shell can be opened more. One could fall back into an over-protective attitude on the part of the patient, even if he has enough regained qualities to be able to decide and live independently again. This is not only an ethical requirement towards the patient, but also towards society.

Another pitfall is more economical in nature. This form of care approach is not cheap. We therefore argue that it should only be applied to the most serious forms of SPMI, for instance when the quality of life is seriously compromised. In view of the future, we could look at whether a form of Oyster Care could also be possible in community-based health care. In that case, there would be more continuity in the approach in the event of a dismissal of the care unit with a greater chance of long-term or even lasting success. A health-economic evaluation of the model would be very useful in this regard.

A final limitation of this article is that it is not yet sufficiently scientifically grounded. It has grown bottom up from the practical experiences of caregivers and professionals. It will be a challenge to better define the group that qualifies for this care approach, to develop measuring instruments for wellbeing and to actually demonstrate in a next step that the quality of life of this patient population increases by applying Oyster Care. This also includes the challenge to involve the patients themselves and their families in the further development of and research into the model, despite the difficulties associated with qualitative research in this target group (43).

### Recommendations

In order to develop Oyster Care, we formulate some recommendations. First, the caregivers involved must promote scientific research among SPMI patients, both quantitative empirical research and qualitative conceptual research. It is important to know exactly how many patients are involved and where they end up. It is also necessary to identify the care needs to improve their wellbeing. This also requires the development of adequate measuring instruments, despite the challenges this setting poses to their development. Measuring tools are even more important when verbal communication is difficult. Based on this research and the existing experience, it is imperative that a specific and specialized training course for Oyster Care is established. This care can only be applied consistently if all caregivers are well-trained. This training has a permanent nature and is continued through supervision and intervision in the workplace. It is important to take a critical look at how Oyster Care works so that it can continue to improve.

The next step is to convince the policymakers. Given the historical development of care, very few financial resources are currently allocated to this patient group. They are, however, very vulnerable patients with high and complex care needs. If caregivers can prove this, using scientific research, they can plead with policymakers for more resources to better develop Oyster Care. In anticipation of these resources, the solidarity within the care units and hospital can also be called upon: the occupancy rate of an Oyster Care unit can temporarily be kept lower or the occupancy rate of the staff can momentarily be increased. Leadership is also very important for the organization of Oyster Care: moral courage to give freedom to the caregivers and to make choices in policy.

Finally, it is imperative to try out this approach on a larger scale and to study it on a broader basis. It is therefore necessary that more researchers study this new concept of care. In this way, it can lead to a broader and more well-founded movement in the care of SPMI patients. This article is only the beginning of a new outlook on SPMI patient care.

## Data Availability Statement

The original contributions presented in the study are included in the article/supplementary material, further inquiries can be directed to the corresponding author.

## Author Contributions

All authors agree to be accountable for the content of the work.

## Conflict of Interest

The authors declare that the research was conducted in the absence of any commercial or financial relationships that could be construed as a potential conflict of interest.
